# Optimization of a fluorescent-mRNA based real-time assay for precise kinetic measurements of ribosomal translocation

**DOI:** 10.1080/15476286.2021.1913312

**Published:** 2021-05-03

**Authors:** Changil Kim, Mikael Holm, Chandra Sekhar Mandava, Suparna Sanyal

**Affiliations:** Department of Cell and Molecular Biology, Uppsala University, Uppsala, Sweden

**Keywords:** Ribosome, protein synthesis, translocation, pyrene mRNA, N-acetyl Phe-tRNA, EF-G, GTP hydrolysis

## Abstract

Kinetic characterization of ribosomal translocation is important for understanding the mechanism of elongation in protein synthesis. Here we have optimized a popular fluorescent-mRNA based translocation assay conducted in stopped-flow, by calibrating it with the functional tripeptide formation assay in quench-flow. We found that a fluorescently labelled mRNA, ten bases long from position +1 (mRNA+10), is best suited for both assays as it forms tripeptide at a fast rate equivalent to the longer mRNAs, and yet produces a large fluorescence change upon mRNA movement. Next, we compared the commonly used peptidyl tRNA analog, N-acetyl-Phe-tRNA^Phe^, with the natural dipeptidyl fMet-Phe-tRNA^Phe^ in the stopped-flow assay. This analog translocates about two times slower than the natural dipeptidyl tRNA and produces biphasic kinetics. The rates reduce further at lower temperatures and with higher Mg^2+^ concentration, but improve with higher elongation factor G (EF-G) concentration, which increase both rate and amplitude of the fast phase significantly. In summary, we present here an improved real time assay for monitoring mRNA-translocation with the natural- and an N-Ac-analog of dipeptidyl tRNA.

## GRAPHICAL ABSTRACT

**Figure:**
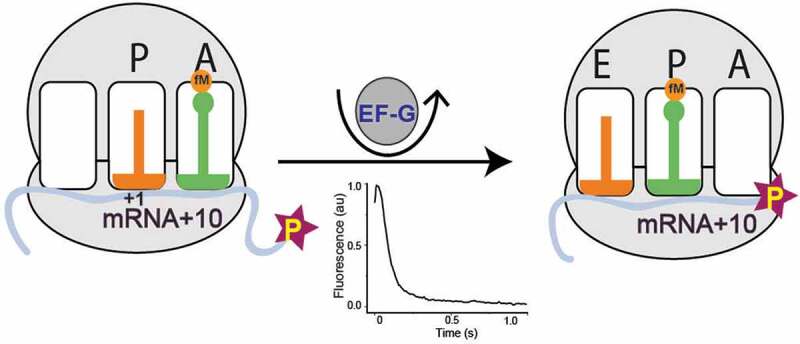

## Introduction

Protein synthesis by the ribosome, during which the genetic information encoded in messenger RNA (mRNA) is translated into proteins, is central to all cellular life. In living cells, ribosomes spend most of their time in peptide chain elongation and the average elongation rate is closely tied to the growth rate of the cell. Therefore, understanding the mechanism of peptide elongation has been a central focus of the ribosome field since the early days of protein synthesis research.

Elongation of the polypeptide chain occurs at a high rate in cells, of about 10–20 amino acids per second per ribosome [1]. The elongation cycle comprises two major events, peptide bond formation and ribosomal translocation. The first step involves elongation factor Tu (EF-Tu), which delivers the aminoacyl tRNAs to the A site and the second step involves elongation factor G (EF-G), which translocates codon-anticodon paired mRNA and tRNAs from A- to P site and P- to E site on the ribosome. In an *in vitro* translation system, fully reconstituted with purified translation components from *Escherichia coli*, the mean time for tripeptide formation starting from the 70S initiation complex (70S IC) has been determined almost as short as *in vivo*, 110 – 150 ms [2,3]. The process includes binding of elongation factor Tu (EF-Tu)•aminoacyl tRNA•GTP ternary complex (TC) to the 70S IC; first peptide bond formation; elongation factor G (EF-G)–mediated ribosomal translocation; binding of the second TC and formation of the second peptide bond. By subtracting the time taken for two peptide bond formations (starting from the binding of TC) from the total time of tripeptide formation, the mean time for EF-G driven ribosomal translocation can been estimated [2,3]. This includes multiple sub-steps of EF-G cycle such as EF-G binding, GTP hydrolysis, mRNA-tRNA movement, ribosomal rearrangements, and EF-G release – occurring in a sequential manner. There are individual assays for monitoring some of the sub-steps listed here, such as fluorescently labelled mRNA or tRNA based assay to follow mRNA-tRNA movement in stopped-flow [4–9] and FRET-based assays for monitoring ribosomal dynamics [10–14] during translocation. However, the components and conditions used in these assays as well as in the functional assay of ribosomal translocation (e.g. tripeptide formation) often differ, which makes it challenging to obtain relatable rates from these assays.

In 2003, Studer et al. first developed a fluorescence based real-time kinetic assay to monitor mRNA movement during ribosomal translocation, by using short, synthetic mRNAs labelled with pyrene at the 3ʹ end [4]. The assay starts by rapid mixing of EF-G to a pre-translocation (pre-T) ribosomal complex programmed with a 3ʹ pyrene labelled mRNA and the peptidyl-tRNA analog N-acetylated Phe-tRNA^Phe^ (NAc-Phe-tRNA^Phe^) placed in the ribosomal A site. During mRNA translocation, the mRNA moves by one codon and as a result the pyrene residue enters in the mRNA channel or moves closer to the ribosome. This change in the environment of the pyrene dye reduces its fluorescence emission. Thus, mRNA movement can be followed directly in real-time by monitoring the change in pyrene fluorescence using a stopped-flow equipped with a fluorescence detector. Due to simple design and the ease of operation, this assay has been popular in kinetic studies related to ribosomal translocation [4,5,11,15–28]. However, this assay, originally referred to as ‘mRNA translocation’ assay [4,5], cannot reflect on the post-mRNA movement steps including ribosomal rearrangement and EF-G release, which can in fact take longer time than the mRNA movement during one cycle of elongation. Thus, calibration of the fluorescence based assay for mRNA movement with the functional assay for translocation by tripeptide formation is crucial for obtaining relatable rates in these two powerful assays, which together can elucidate the mechanism and the limiting factors of ribosomal translocation.

**Figure 1.**
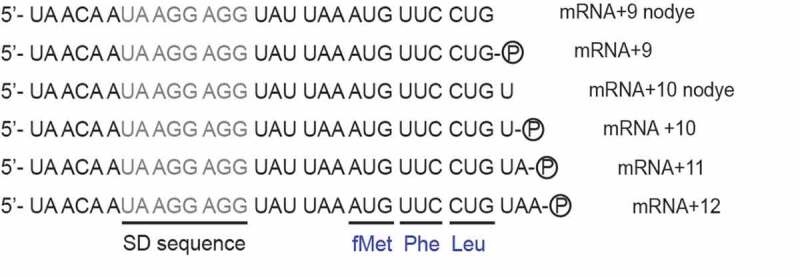
Sequence of the mRNAs without or with the pyrene dye (the circled P) attached covalently to the 3ʹ end. The mRNAs include SD sequences (grey colour) and code for a tripeptide fMet-Phe-Leu. The A of the start codon AUG is counted as +1. The mRNA names are based on the number of nucleotides starting from the +1 position

In the original study, an mRNA labelled with pyrene, nine bases downstream of the start codon (mRNA+9) was proposed as the best mRNA for this assay [4] based on the fact that it produced the largest change in the fluorescence signal upon translocation. However, recent results from our laboratory indicate that mRNA+9 is almost two-fold slower in translocation than longer, unlabelled mRNAs, determined by the tripeptide formation assays using quench-flow [2]. Therefore, our primary aim in this work is to identify an mRNA which produces a high-fluorescence signal in the stopped-flow based mRNA movement assay, while simultaneously undergoing a complete elongation cycle, measured by tripeptide formation in quench-flow, with rates comparable to those of longer unlabelled mRNAs. To this end, we systematically characterized pyrene-labelled mRNAs of different lengths (+9 to +12; Fig. 1) in a tripeptide formation assay in quench-flow in parallel to the fluorescent-mRNA based stopped-flow assay. Our careful comparison identified mRNA+10, one base longer than the commonly used mRNA+9 (Fig. 1), as the most suitable mRNA for obtaining physiologically relevant kinetic rates for translocation. Not only did mRNA+10 produce the second-highest fluorescence change in stopped-flow, it simultaneously showed fast tripeptide-formation kinetics – comparable with longer mRNAs. We have further optimized the conditions using the popular the peptidyl-tRNA analog N-acetylated Phe-tRNA^Phe^ (NAc-Phe-tRNA^Phe^) in the stopped-flow based assay. This analog translocates significantly slower than the natural peptidyl tRNAs and produces biphasic kinetics [24,25,29], making rate analysis challenging. By varying temperature, and titrating Mg^2+^, EF-G and NAc-Phe-tRNA^Phe^ in the reaction, we have successfully derived the best conditions for obtaining a predominant fast phase (~90%) in this assay. These results are not only important for identifying the potential reason for the biphasic kinetics, but also for explaining disparities in results obtained from this assay in the literature.

## Materials and methods

### Components and buffer preparation

All his-tagged translation factors (IF1, IF2, IF3, EF-Tu, EF-Ts and EF-G) and Leu and Phe aminoacyl tRNA synthetases (LeuRS and PheRS) (in-house laboratory clones) were over expressed in *E. coli* BL21(DE3) cells and purified using affinity chromatography on Ni-IMAC column from GE Healthcare [30,31]. 70S ribosomes (*E. coli* MRE600) and f[^3^H]Met-tRNA^fMet^ were prepared according to Antoun et. al. [32]. NAc-Phe-tRNA^Phe^ was prepared as described by Haenni et al, 1966 with minor modifications [33].

Six mRNAs were designed similar to those in Studer et al. [4] (Fig. 1) and purchased from IBA GmbH, Germany. All mRNAs used in this study contain a strong Shine-Dalgarno sequence (UAAGGAGG) and a small open reading frame encoding the peptide sequence Met-Phe-Leu. The open reading frame (underlined in Fig. 1) was followed by the 3ʹ end either immediately (mRNA+9 and mRNA+9 nodye) or after one, two or three additional nucleobases (mRNA+10, mRNA+10 nodye, mRNA+11 and mRNA+12). All mRNAs, except those where ‘nodye’ is mentioned in the name, had a pyrene fluorophore covalently attached to the 3ʹ end with a short carbon linker (IBA GmbH, Germany).

All experiments were performed in HEPES-polymix buffer, pH 7.5 (5 mM HEPES (pH 7.5), 100 mM KCl, 5 mM NH_4_Cl, 0.5 mM CaCl_2_, 5 mM Mg(CH_3_COO)_2_, 8 mM putrescine, 1 mM spermidine and 1 mM dithioerythritol) at 37°C. The reaction buffers contained energy pump components ATP (1 mM), GTP (1 mM), phosphoenolpyruvate (PEP, 10 mM), pyruvate kinase (PK, 50 µg/ml), myokinase (MK, 2 µg/ml). Addition of ATP and GTP led to the free Mg^2+^ concentration 2 mM, which is close to the physiological range in bacteria *E. coli* [34]. PK and MK were not added in translocation assay with NAc-Phe-tRNA^Phe^. ATP and GTP were purchased from GE Healthcare. ^3^H-Met was from Perkin-Elmer. All other chemicals and reagents were from Sigma-Aldrich.

### Di- and tripeptide formation assays using quench-flow

For measuring f[^3^H]Met-Phe dipeptide formation with time, mix A containing 70S ribosome (1 µM), initiation factors (IF1, IF2, IF3, 2 µM each), f[^3^H]Met-tRNA^fMet^ (1.5 µM), MFL mRNA(s) (1.2 µM) (Fig. 1), and mix B containing EF-Tu (10 µM), EF-Ts (1 µM), tRNA^Phe^ (4 µM), Phe (200 µM), PheRS (0.5 µM) were incubated separately at 37°C for 15 min. The reaction was started by rapid mixing of equal volume of both mixes in a Quench-flow (RQF 3-KinTek Corporation). The reactions were quenched at different time points using formic acid (17% final). The samples were treated with 0.5 M KOH, to release the peptides from the tRNAs. The relative amounts of f[^3^H]Met and f[^3^H]Met-Phe were determined by Reverse Phase-High Performance Liquid Chromatography (RP-HPLC) (C18 column) with on-line radiation detection as described by Holm *et al*. [3]. The dipeptide formed over time was fitted to a single exponential function ($\left(y=y_{0}+Ae^{-kt}\right)$, where *k* corresponds to the apparent rate constant and *A* to the total amplitude. The mean time for the first peptide bond formation starting from binding of TC and including entire EF-Tu cycle, (τ_p1_), was determined as 1/*k*.

In order to follow the f[^3^H]Met-Phe-Leu tripeptide formation starting from the 70S IC, the reaction mixes were prepared as above with addition of EF-G (10 µM), tRNA^Leu1^ (4 µM), Leu (200 µM) and Leu-synthetase (0.5 µM) to the mix B. For conducting the reaction and to analyze the product, the same procedure as the dipeptide experiment was followed. The fraction of tripeptide formed (f[^3^H]Met-Phe-Leu) was fitted with a model equation composed of three consecutive irreversible steps as previously described [2,3]. The total mean time for tripeptide formation (τ_tripeptide_) was obtained by calculating the reciprocal of the rate constants.

For determination of the mean time of the second peptide bond formation (τ_p2_) starting from binding of the second TC in a tripeptide reaction, three mixes were prepared. The mixes A and B were prepared with 2X concentration of all components used in the dipeptide reaction. The mix B also contained 2 µM EF-G. The mix C contained EF-Tu (5 µM), EF-Ts (1 µM), EF-G (9 µM), Leu (200 µM), Leu-synthetase (0.5 µM), tRNA^Leu1^ (4 µM). After individual incubation at 37°C for 15 minutes, one volume of each of A and B were manually mixed, which would result in formation of f[^3^H]Met-Phe-tRNA^Phe^ and translocate it to the P site. To this, two volumes of the mix C were rapidly added in quench-flow. The quenching of the reaction and the separation of the peptides using RP-HPLC was done as described above. The fraction of tripeptide, f[^3^H]Met-Phe-Leu formed over time was fitted with a single exponential function in order to determine τ_p2_.

**Figure 2.**
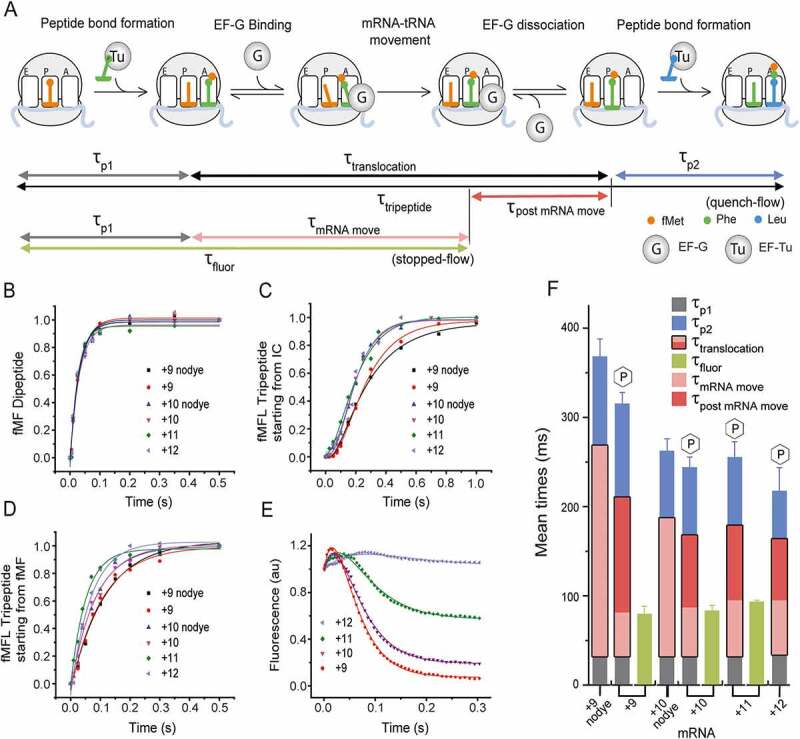
Determination of the optimal length of the mRNA for fast rates and large fluorescence change

The mean time of EF-G cycle referred here as translocation, τ_translocation_, was calculated by subtraction of τ_p1_ and τ_p2_ from τ_tripeptide_ as shown in Fig. 2A and Table 1 [2]. The final rate constants and the corresponding mean times were obtained by averaging the individual values obtained from at least three independent experiments and expressed with standard deviation.

**Table 1.** The mean time of different steps of elongation starting from either 70S IC or pre-T complex measured by quench-flow and stopped-flow

A. Reaction starting from 70S IC, the elongation mix contains natural peptidyl tRNA							
	Quench-flow measurement			Stopped-flow measurement	Derived mean times following the model shown in Figure 2A		
mRNA	τ_p1_ (ms)	τ_tripeptide_ (ms)	τ_p2_ (ms)	τ_fluor_ (ms)	τ_translocation_ (τ_tripeptide –_ (τ_p1_ + τ_p2_)) (ms)	τ_mRNA mov_ (τ_fluor –_ τ_p1_) (ms)	τ_post mRNA move_ (τ_translocation –_ τ_mRNA mov_) (ms)
MFL+9 nodye	32 ± 2	368 ± 20	100 ± 6	-	236 ± 15	-	-
MFL+9	30 ± 2	315 ± 13	119 ± 15	79 ± 9	180 ± 18	49 ± 9	131 ± 9
MFL+10 nodye	32 ± 3	261 ± 15	75 ± 6	-	154 ± 16	-	-
MFL+10	31 ± 3	244 ± 12	76 ± 10	83 ± 6	137 ± 16	52 ± 7	85 ± 16
MFL+11	31 ± 3	255 ± 18	66 ± 8	93 ± 2	158 ± 20	62 ± 4	96 ± 20
MFL+12	33 ± 3	217 ± 27	56 ± 6	-	128 ± 28	-	-
B. Reaction starting from pre-TC with NAc-Phe- tRNA^Phe^ in the A sitef							
	Quench-flow measurement	Stopped-flow measurement					
mRNA	τ_translocation_ + τ_p2_ (ms)	τ_mRNA mov_ (ms)					
MFL+9	2381 ± 227	112 ± 6					
MFL+10	1667 ± 139	119 ± 7					

**Figure 3.**
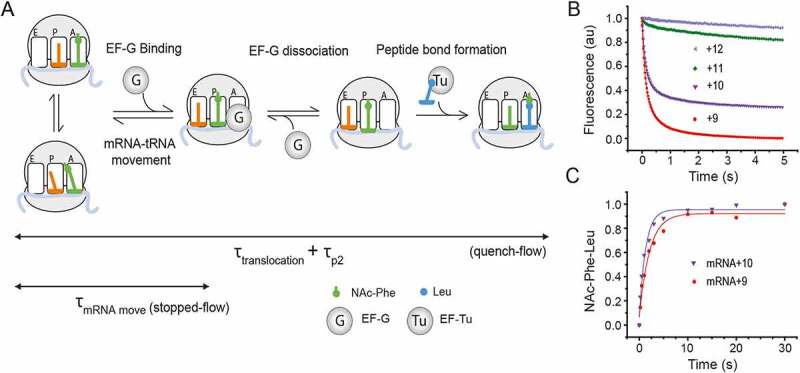
Kinetics of translocation of the pre-T complex containing NAc-Phe-tRNA^Phe^ and pyrene labelled mRNAs

*See* Materials and Methods and Figures 2A and 3A for derivation of the mean time parameters. The results are average of minimum three identical replicates with standard deviation.

(A) Schematic representation of the peptide elongation cycle on the ribosome starting from the 70S IC. The process includes two peptide bond formation steps and one translocation step driven by EF-G. The di- and tripeptide formation experiments were conducted in quench-flow, where an elongation factor mix containing the respective TCs (5 µM) was rapidly mixed with the 70S IC (0.5 µM) containing mRNA and fMet-tRNA^fMet^ in the P site. By fitting the kinetic data as described in the Materials and Methods, the mean times of the first peptide bond formation (τ_p1_), second peptide bond formation (τ_p2_) and tripeptide formation (τ_tripeptide_) were determined. Both τ_p1_ and τ_p2_ include time starting from binding of TC and entire EF-Tu cycle. The mean time of a full translocation reaction (τ_translocation_) was calculated as [τ_tripeptide_– (τ_p1_+ τ_p2_)]. In parallel, the kinetics of translocation starting from the 70S IC was followed in stopped-flow, where the fluorescence change of the 3ʹ pyrene labelled mRNAs was monitored in real time. The mean time τ_fluor,_ indicated total time of all events starting from the 70S IC up to and including mRNA movement. The mean time for mRNA movement, τ_mRNA move_, was determined by subtracting τ_p1_ from τ_fluor_. (B) Kinetics of the first peptide bond formation and (C) tripeptide formation in quench-flow starting from the 70S IC with the mRNAs as indicated.(D) Kinetics of the second peptide bond formation starting from the post-translocation complex with fMet-Phe-tRNA^Phe^ in P site. (E) Kinetics of EF-G mediated mRNA movement during translocation in a stopped-flow starting from the 70S IC, monitored by the changes in pyrene fluorescence (343 nm excitation, 360 nm long-pass filter) with the mRNAs.(F) Bar diagram for direct comparison of the mRNAs for the mean times of different steps of elongation. The three stacked bars indicate τ_tripeptide_ obtained by quench-flow with clear demarcations for τ_p1_ (grey), τ_translocation_ (pink/red) and τ_p2_ (blue). For fluorescent mRNAs τ_translocation_ (pink/red) is divided into τ_mRNA mov_ (pink) and τ_post mRNA move_ (red). For mRNAs without dye entire τ_translocation_ is in pink. The stopped-flow based mean times τ_fluor_ are presented by the green bars. The error bars represent standard deviation.

### Ribosomal translocation in stopped-flow with pre-T complex containing natural dipeptidyl tRNA in the A site

Mixes A and B, same as in the quench-flow experiments for tripeptide formation starting from the 70S IC, were formed and incubated at 37°C for 15 min. Equal volume of A and B were mixed in a stopped-flow instrument (Applied Photophysics) and the fluorescence emission was followed with time using a 360 nm long-pass filter (Comar Optics Ltd). The excitation wavelength was 343 nm, typical for pyrene [4,35]. The fluorescence traces showed an initial small increase followed by a predominant monophasic decrease. The traces were fitted to a double exponential function using the equation $y=y_{0}+A_{1}\left(1-e^{-k_{1}t}\right)+A_{2}\left(1-e^{-k_{2}t}\right)$, where *k*_1_ and *k*_2_ are the apparent rate constants, and *A*_1_and *A*_2_ are respective amplitudes of the first (increasing) and second (decreasing) phases respectively. The τ_fluor_, which includes the mean times for first peptide bond formation (τ_p1_) and the movement of the fluorescent mRNA (τ_mRNA move_), was determined as 1/*k*_1_ + 1/*k*_2_. The τ_mRNA move_ value was then estimated by subtracting τ_p1_ obtained from the quench-flow experiments from τ_fluor_ (Table 1). It should be noted that τ_mRNA move_ is shorter than τ_translocation_. This is likely due to the fact that τ_translocation_ includes also time for ribosomal rearrangement and EF-G release prior to next EF-Tu TC binding and the mRNA-fluorescence based assay cannot detect those. It is unlikely that the time gap indicates any event before mRNA movement as τ_p1_ includes peptide bond formation, which happens after EF-Tu release and immediately prior to EF-G binding. Therefore, we indicate the time gap between τ_translocation_ and τ_mRNA move_ as τ_post mRNA move_, which is the mean time for the post mRNA movement steps. τ_post mRNA move_ was estimated by subtracting τ_mRNA move_ from τ_translocation_ (Table 1). The experiments were repeated at least three times and the average rate constants with standard deviation were determined.

### Ribosomal translocation in stopped-flow and quench-flow with pre-T complex containing NAc-Phe-tRNA^Phe^

Ribosomal translocation and peptidyl transfer experiment with different length of mRNAs were also carried out starting from pre-T complex with peptidyl tRNA analog NAc-Phe-tRNA^Phe^ in A site and deacylated tRNA^fMet^ in the P site. The pre-T complex was formed by first incubating 1 µM 70S with 1.2 µM MFL coding mRNA and 2 µM tRNA^fMet^ at 37°C for 15 min, followed by 20 min incubation with addition of 10 µM NAc-Phe-tRNA^Phe^.

The pre-T complex (1 µM) was mixed with EF-G (10 µM) in a stopped-flow and the change in pyrene fluorescence was followed as described above. As the traces were biphasic, they were fitted with the double exponential function, $y=y_{0}+A_{1}\left(1-e^{-k_{1}t}\right)+A_{2}\left(1-e^{-k_{2}t}\right)$. τ_mRNA move_ was derived from the rate of the major phase, *k*_1_ in most of the cases.

For estimation of the mean time of the complete elongation cycle (τ_translocation_ + τ_p2_, see the scheme in Fig. 3A), equal volumes of the pre-T complex (1 µM) and the elongation mix (10 µM EF-G and 2 µM Leu TC comprising [^3^H]Leu-tRNA^Leu1^) were rapidly mixed in a quench-flow and NAc-Phe-[^3^H]Leu formation was determined by separating the peptides in RP-HPLC as described above. The data are fitted with a single exponential function.

(A) Schematic representation of the peptide elongation cycle on the ribosome starting from the pre-equilibrated pre-T complexes, which are in equilibrium between the classical and the hybrid state. EF-G binding and GTP hydrolysis lead to mRNA movement by one codon. The ribosome complex undergoes certain structural rearrangements and EF-G releases, thereby leaving the ribosome ready for the next peptide bond formation. The meantime of the whole elongation cycle (τ_translocation_ + τ_p2_) was determined by rapidly mixing NAc-Phe-tRNA^Phe^ containing pre-T complex (0.5 µM) with EF-G (5 µM) and Leu TC (1 µM) in a quench-flow and following the formation of NAc-Phe-Leu against time. In parallel, the kinetics of the mRNA movement was followed in stopped-flow, where EF-G (5 µM) was rapidly mixed to a NAc-Phe-tRNA^Phe^ containing pre-T complex (0.5 µM). As a result of the mRNA movement, the fluorescence of the 3ʹ pyrene labelled mRNAs decreases, which is monitored in stopped-flow against time. The τ_mRNA move_ was determined from the reciprocal of the rates obtained from the fluorescence traces.(B) Kinetics of mRNA movement upon EF-G binding to the pre-T complexes containing NAc-Phe-tRNA^Phe^ and pyrene labelled mRNAs: mRNA+9 (red circle), mRNA+10 (violet inverted triangle), mRNA+11 (green diamond) and mRNA+12 (light purple left triangle). The pyrene fluorescence was monitored with 360 nm long-pass filter (343 nm excitation).(C) Kinetics of NAc-Phe-Leu formation in a quench-flow by rapid mixing of a pre-T complex with EF-G and Leu TC.

The translocation assay with pre-T complex was conducted in stopped-flow by varying (i) the mRNAs (Fig. 1); (ii) NAc-Phe-tRNA^Phe^ concentration; (iii) EF–G concentration (0.5–10 µM); (iv) temperature (20, 25, 30 and 37°C), (v) Mg^2+^ concentration by adding additional 1–10 mM Mg(CH_3_COO)_2_. Further, the translocation assays with pre-T complex were also conducted in quench-flow to compare the mRNAs.

## Results

### The mRNA+10 translocates at the same rate as the longer mRNAs

All six mRNAs listed in Fig. 1 were subjected to dipeptide (fMet-Phe) and tripeptide (fMet-Phe-Leu) formation assay in quench-flow. The tripeptide formation assays were conducted starting from (i) the 70S IC and (ii) 70S post-translocation complex containing dipeptidyl tRNA in the P site. The mean times for formation of the first peptide bond (τ_p1_), determined from the dipeptide assay were identical (~31 ms) for all mRNAs. This indicates that neither mRNA length nor pyrene labelling had any effect on this reaction (Fig. 2B, grey bars in Fig. 2F, Table 1A). The mean time of fMet-Phe-Leu tripeptide formation (τ_tripeptide_), and the mean time of formation of the second peptide-bond (τ_p2_), however, varied for the mRNAs, with unlabelled mRNA+9 (mRNA+9 nodye, Fig. 1) and pyrene-labelled mRNA+9 being the slowest ones (Fig. 2C, Fig. 2D, Fig. 2F, Table 1A). τ_tripeptide_ and τ_p2_ for mRNA+9 were 315 ± 13 ms and 119 ± 15 ms, respectively. These mean times were at least 70 and 45 ms longer than those for the longer mRNAs. In contrast, the τ_tripeptide_ and τ_p2_ values for mRNA+10 were 244 ± 12 ms and 76 ± 10 ms, respectively, similar to those for the longer mRNAs (Table 1A). When the unlabelled mRNAs were compared, mRNA+9 nodye was significantly slower than mRNA+10 nodye, in both tripeptide and second peptide bond formation (Fig. 2C, Fig. 2D, Fig. 2F, Table 1A).

The mean time of translocation (τ_translocation_) was determined by subtracting the sum of τ_p1_ and τ_p2_ from τ_tripeptide_. The τ_translocation_ for mRNA+9 was 180 ± 18 ms, while τ_translocation_ for all other mRNAs (including mRNA+10) was ~140 ms, 40 – 50 ms faster than for mRNA+9 (red bars in Fig. 2F, Table 1A). Thus, the mRNA+9 is clearly defective in overall translocation. Comparison of the τ_p2_ values also demonstrate that the mRNA+9 is also defective in the subsequent peptide-bond formation, where it takes about 43 ms longer than mRNA+10 and longer mRNAs. Interestingly, these defects are ameliorated in mRNA+10 by addition of just one additional base at the 3ʹ end of the mRNA+9, which is as fast as the longer mRNAs in these vital steps. For convenience, all rates are summarized in Supplementary Table 1A.

### The mean time of mRNA movement in translocation does not depend on the mRNA length but the mean time of post mRNA movement does

Stopped-flow experiments were conducted by addition of EF-Tu in ternary complex with natural aminoacyl tRNAs and EF-G to 70S IC carrying pyrene-labelled mRNAs of different lengths, to obtain information about both the amplitude and the rate of the fluorescence change induced by mRNA movement during translocation (Fig. 2E). The whole experiment involves several steps, namely binding of the ternary complex, peptide bond formation, EF-Tu release, binding of EF-G and subsequent mRNA movement and translocation. The mRNAs, +9, +10 and +11, showed an initial short increase followed by a prominent single-phase decrease in pyrene fluorescence similar to our previous report [36]. While the monophasic fluorescence decay indicates mRNA movement during translocation, as also suggested by previous studies [4,5,11,16,17,19,20,25,37], the initial fluorescence increase probably happens during the prior events listed above. As explained in the Materials & Methods section, experimental data were fitted with a double exponential function to estimate the τ_fluor_, i.e. the sum of τ_p1_ and τ_mRNA move_ (Table 1A).

In our experiments the mean time of all steps, up to and including mRNA movement (τ_fluor_), was ~80 ms for the +9, +10 and +11 fluorescent mRNAs (purple bars in Fig. 2F, Table 1). Thus, the mean time of mRNA movement (τ_mRNA move_), estimated by subtracting τ_p1_ from τ_fluor_ was ~50 ms for all three mRNAs, thereby suggesting that the mean time of mRNA movement (τ_mRNA move_) is not dependent on the length of the mRNA. The rates are listed in Supplementary Table 1A.

It is clearly noticeable from Table 1 that τ_mRNA move_ is much shorter than τ_translocation_. This means that post mRNA movement steps of translocation, namely ribosomal rearrangement and EF-G release occupy significant fraction of the total time for translocation. The mean time of this step, presented as τ_post mRNA move_ is estimated by subtraction of τ_mRNA move_ from τ_translocation_. This phase is longest for mRNA+9, 131 ± 9 ms. In contrast, the mRNA+10 or longer mRNAs spent about 90 ms in this step. Thus, the mRNA+9 is grossly defective in the post-mRNA movement steps of ribosomal translocation, taking about 40 ms longer than the longer mRNAs.

In agreement with the results obtained by Studer et al. [4], the relative amplitude of the fluorescence change decreased with increasing length of the mRNA. mRNA+9 and mRNA+10 showed the largest and the second-largest fluorescence change, respectively (Fig. 2E). In comparison, mRNA+11 had a roughly 50% smaller fluorescence amplitude and almost no change in fluorescence could be detected with mRNA+12. Hence we conclude that the mRNA+12 or longer mRNAs labelled with fluorescent dye at the 3ʹ are not suitable for this stopped-flow based assay.

Since the mRNA with the largest fluorescence change, mRNA+9, translocated significantly slower than the longer mRNAs and lead to much slower formation of the second peptide-bond (Fig. 2F, Table 1A), functional studies with this mRNA are likely to be compromised. However, mRNA+10 restored the rates of these two steps to comparable magnitude as with longer unlabelled mRNAs, while maintaining near-maximum fluorescence change. Thus, mRNA+10 is undoubtedly the best mRNA for this assay.

### The dipeptidyl tRNA analog NAc-Phe-tRNA^Phe^ is less efficient in translocation than natural dipeptidyl tRNAs

The pre-T complex containing natural peptidyl tRNAs in the ribosomal A site is inherently unstable on the timescale of typical laboratory work. Thus, tRNAs charged with N-acetylated amino acids are commonly used as A-site peptidyl tRNA analogs to mimic the pre-T complex. The binding of such analogs is a factor-independent equilibrium process and the substrates can therefore be supplied in large excesses [4,5,11,16,17,19,20,25,37]. We designed a set of experiments to compare translocation of a pre-T complex formed by pre-equilibration with a commonly used peptidyl tRNA analog NAc-Phe-tRNA^Phe^ to a pre-T complex containing natural fMet-Phe-tRNA^Phe^, formed as described above, in continuous progression (without pre-equilibration), starting from the 70S IC (Fig. 3A).

First we conducted the stopped-flow based assay using the pyrene-labelled mRNAs. Irrespective of the length of the mRNAs (+9 to +12), translocation of NAc-Phe-tRNA^Phe^ produced a biphasic fluorescence-decay curve. The fast phase accounted for 80–90% of the amplitude and the slow phase accounted for the remaining 10%– 20% (Fig. 3B, Supplementary Table 2). Contrary to the reactions with natural dipeptidyl tRNAs (Fig. 2E), no initial increase in fluorescence could be seen (Fig. 3B). Our biphasic decay curves were similar to previous reports [5,11,19,20,25]. However, since the fast phase was significantly larger than the slow phase in our assay (Fig. 3B), we estimated τ_mRNA move_ as a reciprocal of the rate *k_1_* (Supplementary Table 2). For both mRNA+9 and mRNA+10, the τ_mRNA move_ with NAc-Phe-tRNA^Phe^ was ~115 ms, which means that under identical reaction conditions, NAc-Phe-tRNA^Phe^ leads to at least two times slower mRNA movement than the natural dipeptidyl tRNA, which takes about 50 ms (τ_mRNA move_ = ~50 ms) (Table 1A). Addition of EF-Tu and EF-Ts in the NAc-Phe-tRNA^Phe^ reaction mix did not change the rate of mRNA movement (Supplementary Figure 1). As shown in Fig. 3B, both mRNA+9 and mRNA+10 produced significant change in pyrene fluorescence upon translocation with NAc-Phe-tRNA^Phe^. In contrast, the mRNA+11 and mRNA+12 produced very small fluorescence changes and therefore accurate translocation rates could not be determined.

**Table 2.** Effect of varying temperature, EF-G and Mg^2+^ concentration, EF-G mutation and GTP analogs on mRNA movement during ribosomal translocation with NAc-Phe-tRNA^Phe.^

	*k_1_*(s^−1^)	*k*_2_ (s^−1^)	A_1_/(A_1_+ A_2_)	τ_mRNA move_ (ms)
A. EF-G (μM)				
0.5	2.8 ± 0.5	0.6 ± 0.05	0.49 ± 0.03	357 ± 67
1.25	4.4 ± 0.1	0.92 ± 0.01	0.76 ± 0.06	227 ± 5
2.5	6 ± 0.2	0.72 ± 0.1	0.84 ± 0.04	167 ± 6
5	8.4 ± 0.5	0.66 ± 0.2	0.88 ± 0.06	119 ± 7
10	10.3 ± 0.3	0.4 ± 0.07	0.91 ± 0.02	97 ± 3
B. Temperature (°C)				
37	8.4 ± 0.5	0.66 ± 0.2	0.88 ± 0.06	119 ± 7
30	3.2 ± 0.1	0.56 ± 0.02	0.74 ± 0.01	313 ± 10
25	1.25 ± 0.06	0.30 ± 0.01	0.62 ± 0.01	800 ± 38
20	0.35 ± 0.07	0.09 ± 0.02	0.47 ± 0.02	2857 ± 571
C. Extra Mg^2+^ (mM)				
1	5.8 ± 1.3	0.71 ± 0.01	0.66 ± 0.01	172 ± 39
2	3.2 ± 0.2	0.43 ± 0.03	0.62 ± 0.03	313 ± 20
3	1.9 ± 0.2	0.28 ± 0.11	0.52 ± 0.02	526 ± 55
5	0.8 ± 0.3	0.17 ± 0.04	0.48 ± 0.05	1250 ± 469
10	0.19 ± 0.01	0.04 ± 0.01	0.49 ± 0.01	5263 ± 277

The biphasic traces of pyrene fluorescence (Figure 5) are fitted with double exponential function. *k*_1_, *k*_2_, and *A*_1_, *A*_2_ are the rate constants and amplitudes of the fast and the slow phases respectively. See *Materials and Methods* for derivation of τ_mRNA move_. The results are average of minimum three experimental replicates with standard deviation.

We also checked NAc-Phe-tRNA^Phe^ in quench-flow for translocation and NAc-Phe-Leu peptide bond formation (Fig. 3C). The length of one elongation cycle (τ_translocation_ + τ_p2_) with NAc-Phe-tRNA^Phe^ was significantly longer than with natural dipeptidyl tRNAs. While τ_translocation_ + τ_p2_ was about 300 ms for the dipeptidyl tRNAs, it was almost 2 sec with NAc-Phe-tRNA^Phe^ (Table 1A, B). Thus, the use of NAc-Phe-tRNA^Phe^ in translocation leads to large functional defects. Interestingly, τ_translocation_ + τ_p2_ is comparatively shorter with mRNA+10 than with mRNA+9, adding further evidence that mRNA+10 is better suited for functional assays than mRNA+9. The rates are reported in Supplementary Table 1B.

### Optimizing the stopped-flow based assay with the peptidyl tRNA analog NAc-Phe-tRNA^Phe^

To identify the optimal conditions for both translocation rate and fluorescence signal amplitude, we systematically titrated NAc-Phe-tRNA^Phe^, EF-G, and Mg^2+^, and varied temperature in the fluorescent-mRNA based translocation assay in stopped-flow.

**Figure 4.**
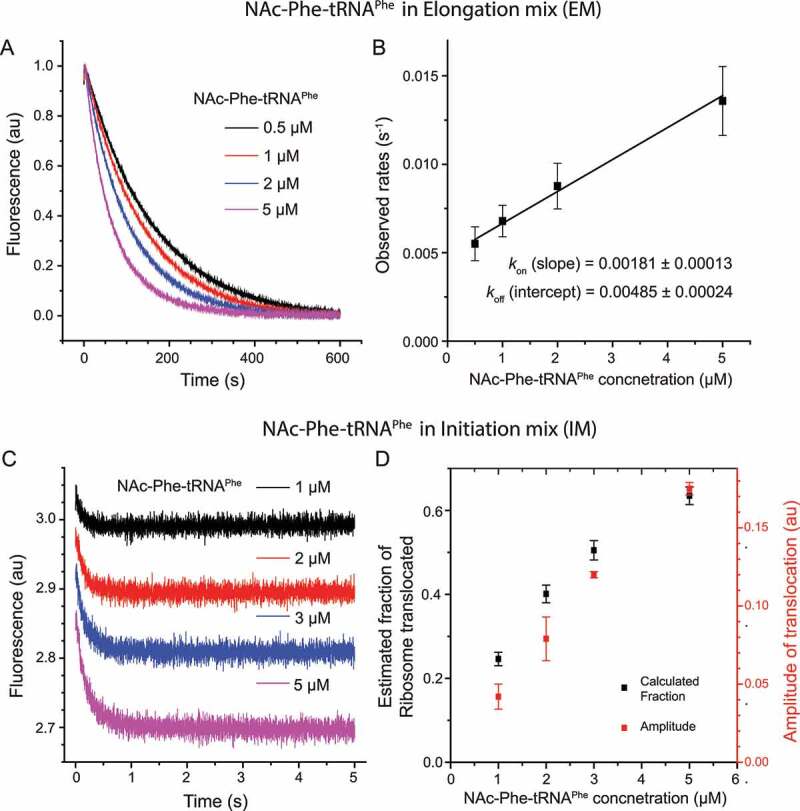
NAc-Phe-tRNA^Phe^ titration in the 3’ pyrene-labelled mRNA+10 based translocation reaction in stopped-flow

First, NAc-Phe-tRNA^Phe^ was titrated by supplying it with the EF-G containing elongation mix such that translocation would require first binding of NAc-Phe-tRNA^Phe^ to the ribosomal A site. Increase in NAc-Phe-tRNA^Phe^ concentration increased the rate of the fluorescence decay in a linear fashion (Fig. 4A) from 0.005 s^−1^ at 0.5 µM to 0.013 s^−1^at 5 µM. As EF-G mediated translocation is much faster (about 8 s^−1^), the rate of the fluorescence change here is limited by association of NAc-Phe-tRNA^Phe^ to the 70S IC. It allowed us to estimate the binding parameter of NAc-Phe-tRNA^Phe^ to the 70S IC. By plotting the observed rates vs. NAc-Phe-tRNA^Phe^ concentrations, the dissociation constant *K_D_* was determined to be 2.7 ± 0.2 µM (Fig. 4B).

(A) Pyrene fluorescence traces obtained by adding NAc-Phe-tRNA^Phe^ (0.5–5 µM) in the elongation mix (EM), which was mixed rapidly with the 70S IC (0.5 µM) carrying mRNA+10 and tRNA^fMet^. (B) The plot of the observed rates of pyrene fluorescence change in (A) versus NAc-Phe-tRNA^Phe^ concentration. The data are fitted with linear equation to obtain the binding parameters. The *K*_*D*_ was determined as *k*_off_/*k*_on_ = 2.7 ± 0.2 µM. (C) Pyrene fluorescence traces obtained by adding NAc-Phe-tRNA^Phe^ (1–5 µM) in the initiation mix (IM) containing 70S IC (0.5 µM), which was mixed rapidly with EM containing EF-G (5 µM). The start point of each fluorescence trace is offset vertically for easy comparison. The data is fitted with double exponential function and presented in Supplementary Table 3. (D) Correlation of the calculated and experimentally determined fractions of the 70S pre-TC, obtained from the *K*_*D*_ value (B) and fluorescence amplitudes (C), respectively. Data represent average of three replicates with standard deviation.

**Figure 5.**
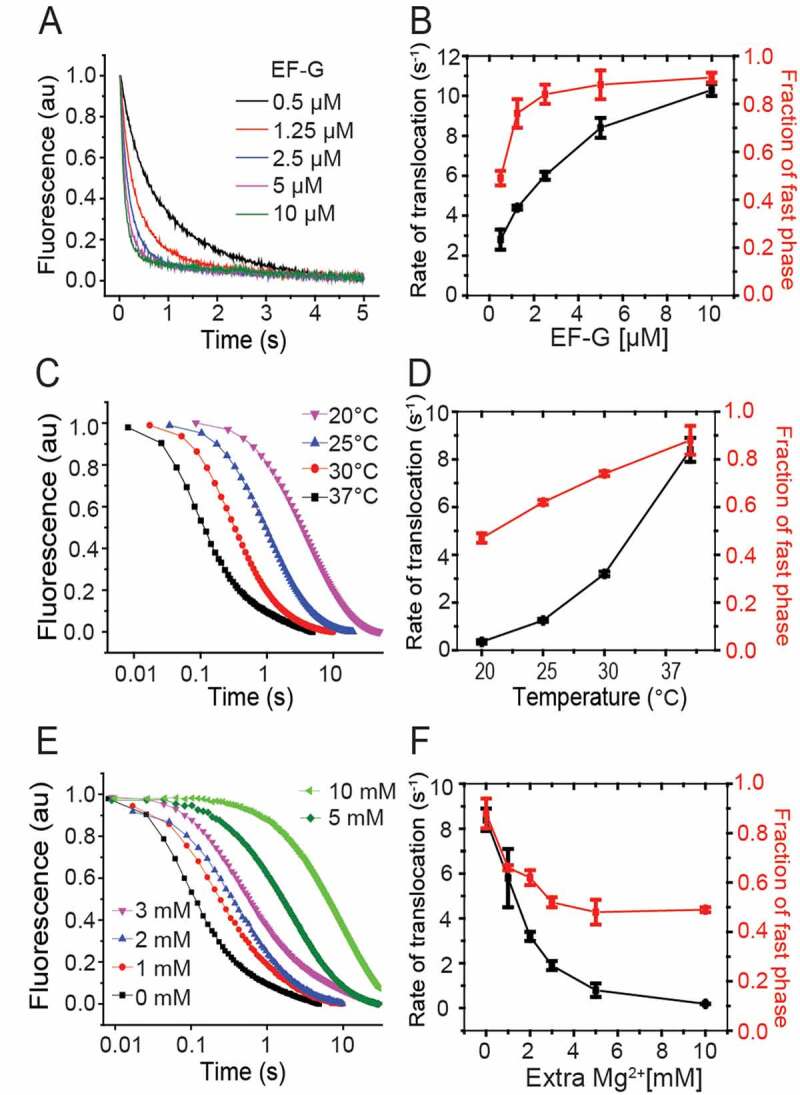
Kinetics of NAc-Phe-tRNA^Phe^ translocation with varying temperature and concentrations of EF-G and Mg^2+^

The kinetics of the mRNA movement during translocation was followed in stopped-flow, where EF-G was rapidly mixed to a NAc-Phe-tRNA^Phe^ containing pre-T complex (0.5 µM). The left panels show the pyrene fluorescence traces monitored with 360 nm long-pass filter (343 nm excitation). The right panels present the rates and amplitudes of the fast phase of the fluorescence traces (Table 2). The plots represent measurements with varying concentration of EF-G (A, B), different temperature (C, D) and varying Mg^2+^ concentration (E, F). The rates and the fractional amplitude of the fast phase are summarized in Table 2.

Next, NAc-Phe-tRNA^Phe^ was titrated (1–5 µM) into the 70S IC mix (0.5 µM), allowing it to pre-equilibrate with the ribosomes to form a pre-T complex. Here, the rates of fluorescence decay were similar over the entire concentration range, but the fluorescence amplitude changed as a function of NAc-Phe-tRNA^Phe^ concentration (Fig. 4C, Supplementary Table 3). The fractions of pre-T complex were directly estimated from the amplitudes of fluorescence traces in Fig. 4C (Supplementary Table 3). These were compared with the fractions of the pre-T complex calculated from the *K_D_* value (Fig. 4B). We observed a substantial correlation between the two, showing that NAc-Phe-tRNA^Phe^ binding to ribosome under this condition is considerably weak (Fig. 4D). On the basis of these results, 2 µM or higher concentration of NAc-Phe-tRNA^Phe^ is required for near-stoichiometric formation of the pre-T complex and un-ambiguous estimation of translocation rates.

Next, EF-G was titrated (0.5 to 10 µM) in the reaction with NAc-Phe-tRNA^Phe^ (Fig. 5A). As expected, translocation was faster at higher EF-G concentrations (Table 2A): τ_mRNA move_ decreased to around 100 ms at 10 µM from 357 ± 67 ms at 0.5 µM of EF-G. Interestingly, the biphasic nature of the fluorescence traces also changed. At 0.5 µM EF-G concentration the amplitude of the fast phase was ~50%, which increased to ~90% with EF-G equal to or more than 5 µM (Fig. 5B, Table 2A). From the rates of translocation at varying EF-G concentrations, the Michaelis-Menten parameters for NAc-Phe-tRNA^Phe^ translocation, (*k_cat_* = 11.9 ± 0.8 s^−1^ and *k_cat_*/*K_M_* = 5.4 ± 1 µM^−1^ s^−1^), were determined (Supplementary Figure 2). The same assay with natural dipeptidyl tRNAs starting from the 70S IC, showed *k_cat_* = 22.8 ± 2.3 s^−1^ and *k_cat_*/*K_M_* = 9.1 ± 0.14 µM^−1^ s^−1^ (Supplementary Figure 2). These results clearly demonstrate that NAc-Phe-tRNA^Phe^ is about two times less efficient than the natural dipeptidyl-tRNAs in EF-G mediated translocation.

Next we studied the effect of temperature and Mg^2+^ ion concentration on the rate of translocation using the pyrene–mRNA assay (Fig. 5C–F). These two parameters have varied a lot in the existing literature [3,4,11,16,25,36,38]. Irrespective of the temperature (20, 25, 30 and 37°C) and Mg^2+^ concentration (1-10 mM Mg^2+^), we obtained biphasic fluorescence traces with varying amplitudes of the fast and the slow phase (Fig. 5C,E, Table 2). As expected, the rates were lower at lower temperatures and higher Mg^2+^ concentrations (Table 2B, C), as also seen by Feldman et al [17,36]. The reactions at 20°C were ~25 times slower than at 37°C (Fig. 5C, Table 2B). Similarly, addition of 10 mM extra Mg^2+^ (at 37°C) slowed the reaction by ~30 times (Fig. 5E, Table 2C). This effect of Mg^2+^ concentration is comparable to its effect on translocation with native tRNAs , as published previously [2,17]. Interestingly, the fractional amplitude of the fast phase (A_1_/(A_1_+ A_2_)) also changed with decreasing temperature and increasing Mg^2+^ (Fig. 5D,F, Table 2B,C). Our results closely match the rates reported in the literature under similar conditions [5,11,16,19,20,25].

## Discussion

The fluorescent-mRNA based assay, originally developed by Studer et al. [4], is popularly used to study ribosomal translocation in real time using stopped-flow [4,5,11,16,17,19,20,25,37]. Our main objective in this work was to optimize this assay by calibrating it with the quench-flow based translocation measurements using unlabelled native substrates.

First, we aimed to identify the optimal length of the pyrene-labelled mRNA that would not only produce a high fluorescence signal, but would also show kinetics comparable to those obtained with longer, natural mRNAs. For that, we tested pyrene-labelled mRNAs of different length (+9 to +12), in the fluorescent-mRNA based assay in stopped-flow, and in parallel, in tripeptide-formation assay in quench-flow. Our results show that mRNA+9, suggested by the original work [4] and used extensively in the literature [4,11,16,17,19,25], is slow in overall translocation and subsequent peptide bond formation (Fig. 2C,D,F and Table 1).Thus, the kinetic rates obtained with mRNA+9 may not be physiologically relevant. However, extending the mRNA by just one more nucleotide rectifies these rate issues. The mRNA+10 translocates as fast as the longer mRNAs and causes no issues with downstream elongation (Fig. 2C,D,F and Table 1). When compared in the fluorescence based stopped-flow assay, all mRNAs moved at similar rates, suggesting that the speed of mRNA movement during translocation is not dependent on the mRNA length (Fig. 2F, Table 1). However, the amplitude of the fluorescence decay showed strong mRNA-length dependence, mRNA+9 being the best one and mRNA+10 being the second best (Figures 2E, Figures 3B). When mRNAs longer than 10 bases were tested, the fluorescence change was small as reported earlier [4], and therefore not suitable for kinetic analysis. Probably for these longer mRNAs, the dye at the 3ʹ end cannot enter the ribosome milieu by one round of translocation. In conclusion, mRNA+10 is clearly the best mRNA for obtaining relatable rates in the fluorescent-mRNA based stopped-flow assay and the quench-flow based tripeptide assay without compromising the fluorescence signal.

When the mRNAs are tested in tripeptide formation assay for determining the mean time of a complete translocation cycle starting from 70S IC, those mRNAs, truncated immediately after the A-site codon (mRNA+9 and mRNA+9 nodye, Fig. 1) show large defects in the post mRNA-movement steps of translocation. These late steps of translocation have recently been implicated in determining both the overall rate [39,40] and possibly accuracy [41] of the process. Most likely, abrupt truncation of these mRNAs leads to problems with ribosomal rearrangement such as reverse swivel of the small-subunit head domain. It is also possible that these truncated mRNAs are partially destabilized in the A site, making the recruitment of the second ternary complex defective and thereby slowing down the second peptide bond formation. It is interesting that addition of just one extra nucleotide, thereby extending the mRNA into the mRNA channel downstream of the A site [42] alleviates these issues. Addition of the pyrene dye at the 3ʹ end also seems to partially improve the situation as labelled mRNA+9 is faster than the unlabelled mRNA+9 in translocation (Fig. 2F, Table 1). These results imply that base interaction in the mRNA channel downstream to the A-site codon is possibly important to anchor the mRNA during translocation.

Another important aspect of this work is that we carefully compared the peptidyl tRNA analog NAc-Phe-tRNA^Phe^ with the natural dipeptidyl tRNA (fMet-Phe-tRNA^Phe^) in the fluorescent-mRNA based translocation assay. The rate of translocation with NAc-Phe-tRNA^Phe^ is about two times lower than with the natural dipeptidyl tRNA (Supplementary Figure 2 and Table 1). This result is not too surprising given that the acetyl group is significantly smaller than an amino acid (f-Met in this case). However, NAc-Phe-tRNA^Phe^ sits quite stably in the ribosomal A-site than the natural dipeptidyl tRNAs, which has half-life of only 10 seconds. This advantage cannot be ignored.

Another notable difference was in the nature of the declining phase of the fluorescence traces. It was clearly biphasic with NAc-Phe-tRNA^Phe^, as also reported by many other studies [5,11,17,19,20,25] (Fig. 4C). In contrast, the natural dipeptidyl tRNAs lead to monophasic fluorescence decay (Fig. 2E) [36]. In NAc-Phe-tRNA^Phe^ translocation assays, increasing temperature led to significantly larger fast phases, while increasing Mg^2+^ had the opposite effect (Fig. 5D, Fig. 5F, Table 2B, C). Increase in the concentration of EF-G also led to predominant (~90%) fast phase (Fig. 5B, Table 2A). Although we cannot determine the exact cause of the differences between the biphasic vs. monophasic curves, we notice that such biphasic curves are frequently reported in the literature, whenever the pre-T complex has been allowed to pre-equilibrate by incubation. This is irrespective of whether the reaction was done with peptidyl tRNA analogs [5,11,19,25,28] or natural peptidyl tRNAs [10,17,38,43]. In contrast, when the pre-T complex was formed by continuous progression from the 70S IC and translocated immediately, the fluorescence decay was monophasic (Fig. 2E) [36]. We suspect, in line with an earlier report [29] that the two phases of the biphasic curves likely represent different translocation rates from the two conformational states of the pre-T ribosome, which are known to populate at equilibrium [13,14,44]. Without pre-equilibrium, the pre-T ribosomes reach a uniform conformational state and therefore show a monophasic transition, a suggestion tentatively supported by recent single-molecule FRET data [45]. EF-G binding likely facilitates transition of the pre-T complex to a translocation-competent state, thereby resulting in near-monophasic kinetics at high concentration.

We also noticed differences in the fluorescence traces obtained with NAc-Phe-tRNA^Phe^ compared to the natural dipeptidyl tRNA. When translocation was initiated by addition of NAc-Phe-tRNA^Phe^ TC and EF-G (in EM) to the programmed 70S IC, an initial short increase of fluorescence could be seen (Fig. 4A). This was absent when translocation was conducted by addition of EF-G to a pre-T complex containing NAc-Phe-tRNA^Phe^ (Fig. 4C). Since the latter did not involve any natural steps for formation of the pre-T complex, we assume that the initial increase in the former case indicates structural rearrangement in the ribosome or the mRNA related to peptide bond formation and EF-Tu release.

The low affinity of NAc-Phe-tRNA^Phe^ for the ribosomal A site during formation of the pre-T complex should also be noted. Binding affinity measurements obtained by titrating NAc-Phe-tRNA^Phe^ into the EF-G mix indicated a fairly high *K_D_* (2.7 ± 0.2 µM) (Fig. 4B). This implies that the low fluorescence amplitude obtained with low concentration of NAc-Phe-tRNA^Phe^ is due to its poor binding to the pre-T complex (Figures 4C, D). However, it is unlikely that the slow phase in translocation with NAc-Phe-tRNA^Phe^ results from this poor affinity, since the rate of binding of NAc-Phe-tRNA^Phe^ was significantly slower (0.013 s^−1^) than the rate of the slow phase (about 0.6 s^−1^) under our experimental condition (Fig. 4A,B).

In summary, this study demonstrates pyrene labelled mRNA+10 as the most suitable mRNA for real-time translocation assay as well as the functional tripeptide formation assay. We further report the best reaction conditions for the fluorescent-mRNA based translocation assay with NAc-Phe-tRNA^Phe^.

## Supplementary Material

Supplemental Material
