# Supplementary material for: Optimization of a fluorescent-mRNA based real-time assay for precise kinetic measurements of ribosomal translocation
Source: RNA Biol. 2021 May 3;18(12):2363–75. doi: 10.1080/15476286.2021.1913312 (PMC8632105; doi:10.1080/15476286.2021.1913312)
Supplement: Supplemental Material [file KRNB_A_1913312_SM1396.pdf]

<u>Supplementary Data</u>

Optimization of a fluorescent-mRNA based real-time assay for precise kinetic measurements of ribosomal translocation

Changil Kim, Mikael Holm, Chandra Sekhar Mandava and Suparna Sanyal*

Department of Cell and Molecular Biology, Uppsala University, Box-596, BMC, 75124, Uppsala

* Corresponding author- Suparna.sanyal@icm.uu.se

Supplementary Table 1. Rates of different steps of elongation starting from either 70S IC or a preformed pre-translocation (pre-T) complex measured by quench flow or stopped flow

A. Reaction starting from 70S IC, the elongation mix contains natural peptidyl tRNA				
	Quench-flow measurement			Stopped-flow measurement
mRNA	k_{p1} (s^{-1})	$k_{tripeptide}$ (s^{-1})	k_{p2} (s^{-1})	k_{fluor} (s^{-1})
MFL+9 nodye	31 ± 2	2.7 ± 0.1	10 ± 0.6	-
MFL+9	33 ± 2	3.2 ± 0.1	8.4 ± 1.1	12.7 ± 1.4
MFL+10 nodye	31 ± 3	3.8 ± 0.2	13.3 ± 1.1	-
MFL+10	32 ± 3	4.1 ± 0.2	13.2 ± 1.7	12 ± 0.9
MFL+11	32 ± 3	3.9 ± 0.3	15.2 ± 1.8	10.8 ± 0.2
MFL+12	30 ± 3	4.6 ± 0.6	17.9 ± 1.9	-

B. Reaction starting from pre-TC with NAc-Phe-$tRNA^{Phe}$ in the A site		
	Quench-flow measurement	Stopped-flow measurement
mRNA	$k_{elongation}$ (s^{-1})	k_{mRNA} (s^{-1})
MFL+9	0.42 ± 0.04	8.9 ± 0.5
MFL+10	0.6 ± 0.05	8.4 ± 0.5

The table contains the rate constants corresponding to the mean time measurements in Table 1. See Figure 2A and 3A for the kinetic scheme and parameter nomenclature.

A. k_{p1}, $k_{tripeptide}$, k_{p2} are the rates for first peptide bond formation, tripeptide (fMet-Phe-Leu) formation, second peptide formation estimated from the quench-flow reactions starting from 70S IC. k_{fluor} is the rate constant obtained from the stopped-flow measurement of translocation with pyrene-labeled mRNA starting from 70S IC.

B. $k_{elongation}$ is the rate of the whole elongation cycle of NAc-Phe-Leu formation in quench-flow starting from the pre-T complex containing NAc-Phe-$tRNA^{Phe}$. k_{mRNA} is the rate for fluorescence change in the pyrene-mRNA fluorescence based stopped-flow assay starting from pre-T complex.

The results are average of minimum three identical replicates with standard deviation.

Supplementary Table 2. Kinetic analysis of mRNA-tRNA translocation starting from pre-T complex with peptidyl tRNA analog NAc-Phe-$tRNA^{Phe}$ in the A site and pyrene-labeled mRNAs of different length.

mRNA	k_1 (s^{-1})	k_2 (s^{-1})	A_1+A_2 (a.u.)	A_1/(A_1+A_2)	$τ_{mRNA}$ (ms)
MFL+9	8.9 ± 0.5	1.33 ± 0.05	1	0.84 ± 0.05	112 ± 6
MFL+10	8.4 ± 0.5	0.66 ± 0.2	0.74 ± 0.02	0.88 ± 0.06	119 ± 7
MFL+11	6.1 ± 0.7	0.24 ± 0.02	0.22 ± 0.04	0.23 ± 0.05	-
MFL+12	1.3 ± 0.8	0.07 ± 0.03	0.16 ± 0.03	0.08 ± 0.02	-

The data corresponds to the Figure 3B and Table 1B. The pyrene fluorescence traces were fitted in double exponential function, k_1 and k_2, and A_1 and A_2, are the rates and amplitudes of the fast and slow phases respectively. $τ_{mRNA}$, is the reciprocal of the major phase k_1. The total amplitude (A_1+A_2) of fluorescence change was normalized against MFL+9. As MFL+11 and MFL+12 produced feeble fluorescence change during translocation, reliable $τ_{mRNA}$ could not be achieved for these two mRNAs. The results are average of three replicates and presented as mean with standard deviation.

Supplementary Table 3. Kinetics of fluorescent mRNA movement as NAc-Phe-$tRNA^{Phe}$ is titrated in the formation of pre-equilibrated pre-translocation complex

NAc-Phe-$tRNA^{Phe}$ concentration (μM)	k_1 (s^{-1})	k_2 (s^{-1})	A_1+A_2 (a.u.)	A_1/(A_1+A_2)	$τ_{mRNA}$ (ms)
1	ND	ND	0.042 ± 0.008	ND	ND
2	8.9 ± 0.7	2.2 ± 0.9	0.079 ± 0.014	0.8 ± 0.1	112 ± 9
3	8.5 ± 0.4	1.4 ± 0.2	0.12 ± 0.002	0.84 ± 0.06	118 ± 6
5	8.4 ± 0.5	0.66 ± 0.2	0.175 ± 0.004	0.88 ± 0.06	119 ± 7

Pyrene fluorescence traces were fitted with double exponential functions; the parameters k_1, k_2, A_1, A_2, and $τ_{mRNA}$ are defined in Materials and Methods. Pre-translocation complexes were formed by incubating 70S ribosomes (0.25 μM), MFL+10 mRNA (0.35 μM) and $tRNA^{fMet}$ (0.5 μM) followed by another incubation after adding the indicated concentration of NAc-Phe-$tRNA^{Phe}$. The results are an average of a minimum of three identical experimental replicates with standard deviation. ND stands for ‘not determined’.

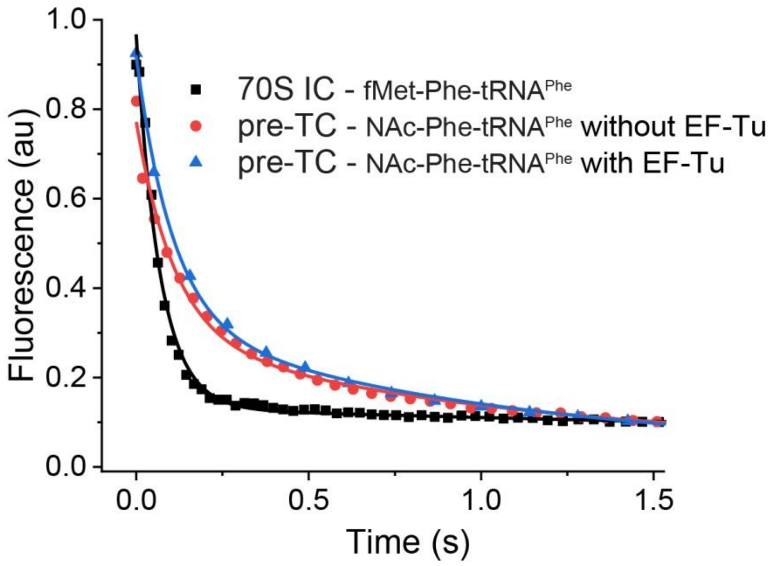

Supplementary Figure 1: Comparision of the time traces of pyrene-mRNA movement on pre-T complex containing natural dipeptidyl tRNA or NAc-Phe-$tRNA^{Phe}$.

The fluorescent mRNA+10 based translocation assay was conducted in stopped-flow with pre-T complex (0.5 µM) containing either fMet-Phe-$tRNA^{Phe}$ or NAc-Phe-$tRNA^{Phe}$. The NAc-Phe-$tRNA^{Phe}$ reactions were conducted with or without EF-Tu and EF-Ts.

The mRNA movement was two times faster with pre-T complex containing natural fMet-Phe-$tRNA^{Phe}$ dipeptidyl tRNA (black square) than with NAc-Phe-$tRNA^{Phe}$ (blue triangle). Addition of EF-Tu and EF-Ts to the NAc-Phe-$tRNA^{Phe}$ reaction (red circle) did not influence the rate.

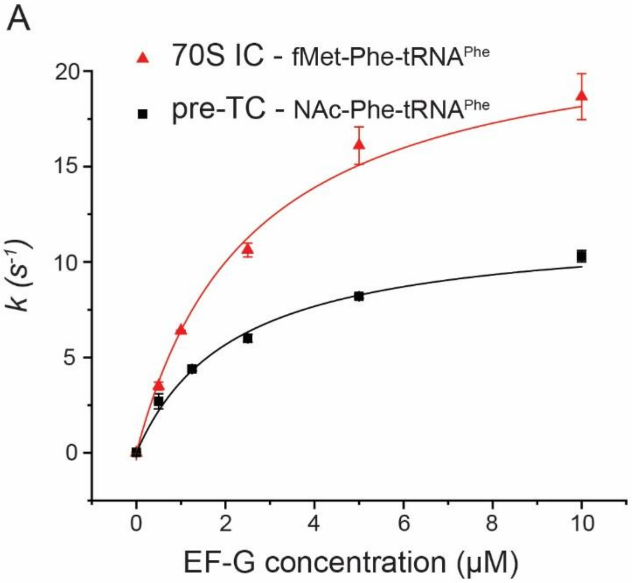

B

	70S IC - fMet-Phe-tRNA^{Phe}	pre-TC - NAc-Phe-tRNA^{Phe}
k_{cat} (s^{-1})	22.8 ± 2.3	11.9 ± 0.8
K_M (µM)	2.5 ± 0.2	2.2 ± 0.4
k_{cat} / K_M ($\mu M^{-1}s^{-1}$)	9.1 ± 0.14	5.4 ± 1

Supplementary Figure 2. Comparison of the kinetic parameters of translocation reactions with natural dipeptidyl tRNA and NAc-Phe-tRNA^{Phe}.

(A) Michaelis-Menten plot of the rates of EF-G mediated translocation, obtained from the pyrene mRNA+10 based stopped-flow assay, starting from two different ribosome complexes. In one case (red triangles) an elongation mix containing EF-Tu TC (Phe-tRNA^{Phe}) and EF-G was added to 70S IC containing fMet-tRNA^{fMet}. In the other case, EF-G was added to a pre-equilibrated pre-T complex containing NAc-Phe-tRNA^{Phe} (black square). (B) Michaelis-Menten parameters estimated from the plots in (A).
